# A bibliometric and visualization-based analysis of temozolomide research hotspots and frontier evolution

**DOI:** 10.3389/fonc.2022.905868

**Published:** 2022-11-15

**Authors:** Peng Song, Hui Li, Kuo Xu, Zi-Wei Li, Xia Ren, Xian-Jun Fu

**Affiliations:** ^1^ College of Pharmacy, Shandong University of Traditional Chinese Medicine, Jinan, China; ^2^ Traditional Chinese Medicine Research Center, Qingdao Academy Shandong University of Traditional Chinese Medicine, Qingdao, China; ^3^ Shandong Engineering and Technology Research Center of Traditional Chinese Medicine, Jinan, China

**Keywords:** temozolomide, glioblastoma, bibliometrics, web of science, development process

## Abstract

The literature related to TMZ research in the Web of Science (WOS) database was analyzed using bibliometrics and visualization by Citespace and VOSviewer.The publication status (number of publications, institutions, and frequency of citations), collaborations, and research focus was analyzed to clarify the current situation of TMZ research. And the recent research on TMZ provides a detailed summary. Based on objective data analysis, this study provides a complete analysis portraying the progression of historical milestones in TMZ development and future research directions from various TMZ research domains.

## Introduction

**Figure 1:**
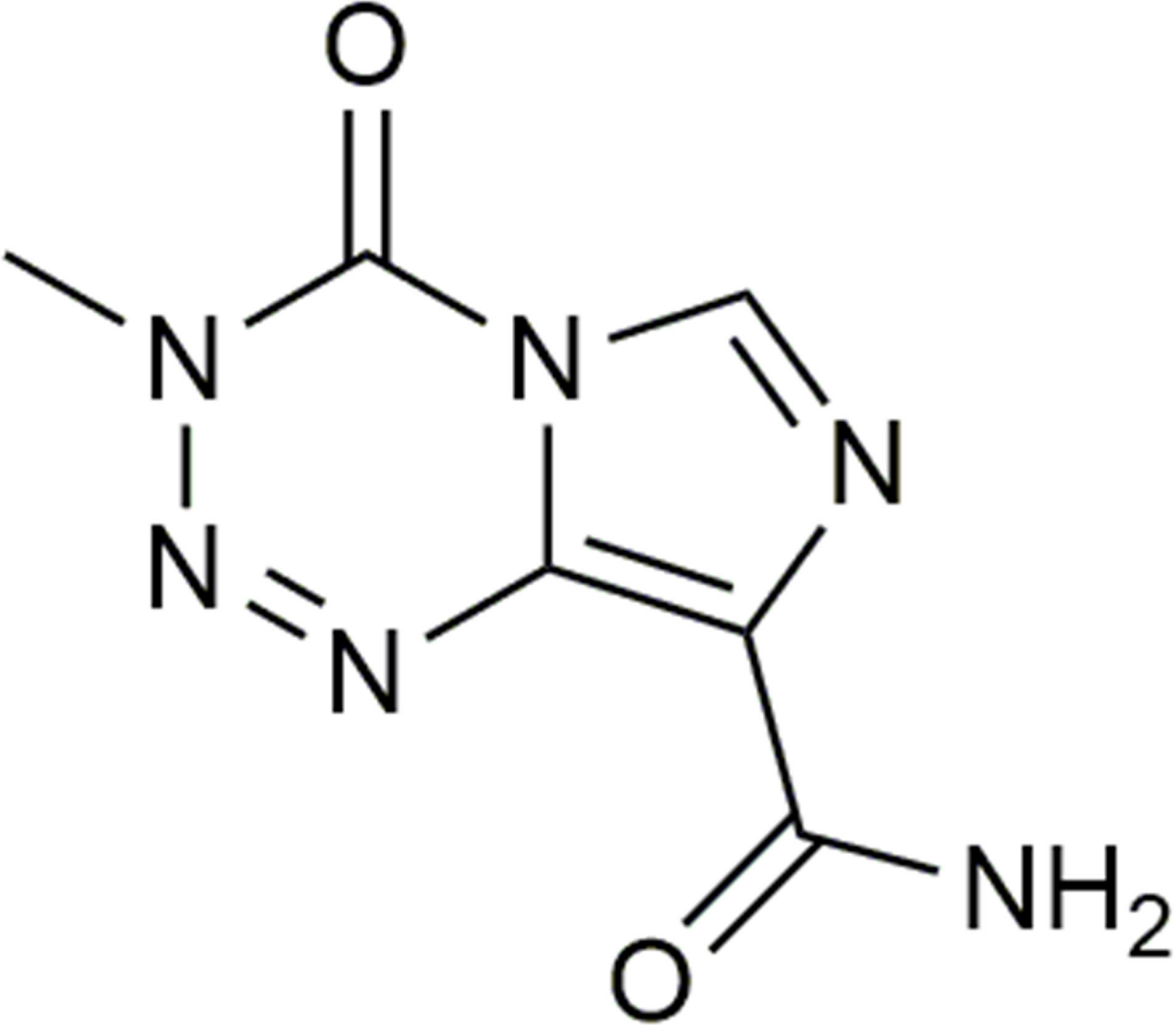
Structural formula of TMZ. Chemical name: 3,4-dihydro-3-methyl-4-oxoimidazo[5,1-d]-as-tetrazine-8-carboxamide.

Temozolomide (TMZ) is a novel imidazole-tetrazine alkylating agent (**Figure 1**). It has a low molecular weight, a broad antitumor range, is lipophilic, and crosses the blood-brain barrier, among other properties. It is rapidly absorbed after oral administration and has no toxicity when combined with other medications (1, 2). On August 11, 1999, the FDA approved TMZ to treat adult recurrent glioblastoma (3), and it has since become the first-line chemotherapeutic medication for malignant glioma and other intracranial tumors (4). TMZ causes tumor cells to die by causing alkylation of the oxygen and nitrogen atoms at position 7 of DNA guanine, resulting in a base mismatch (5).

Most of the research literature on TMZ focuses on studies of resistance mechanisms and clinical trials, and the review literature on TMZ focuses on some specific aspects. It is relatively limited, and there are few overall analyses of TMZ.

Bibliometrics, which combines mathematics, statistics, and bibliography, uses quantitative analysis to investigate the discipline’s structural features and hot trends and evaluate and predict outcomes (6). Publications, authors, keywords, and literature citations are the statistical objects. Using bibliometric software to visualize and analyze relevant literature, as opposed to textual descriptions of traditional theoretical reviews, can present the interrelationships among the literature of a discipline or a research field as a scientific knowledge map, which can not only sort out past research trajectories but also better grasp future research trends and directions (7). Thanks to computer tools, the topic has gotten more attention in terms of theory and practice. Scientific knowledge mapping, which compiles the literature, is commonly employed on this premise. CiteSpace and VOSviewer are two knowledge mapping-focused information visualization and analysis programs.

Only a few cases of knowledge mapping being used to assess the overall state of TMZ research have been reported so far. Based on this, we hope to present the development process and research hotspots in TMZ research in scientific knowledge mapping more intuitively and effectively by using software to data-mine, integrate, and analyze related research literature, and provide a reference for drug development.

## Materials and methods

The Web of Science (Wos) core collection database was selected, indexed by Science Citation Index Expanded (SCI-E), Current Chemical reaction (CCR-E), and Index Chemicus (IC), and “ Temozolomide” was used as the subject to search the relevant literature between 1994 and 2021, and articles and reviews were used as the type of literature, and Citespace was used to de-duplicate and refine the search results, resulting in 12,910 publications. The records were exported as plain text files with the content of “Full records and cited references” for subsequent analysis.

CiteSpace, VOSviewer, and HistCite bibliometric software applications were used to examine the data collected. The data exported from the WOS database were analyzed in this paper using the citation analysis tool HistCite (HistCite Pro version 2.1 recompiled; Dr. Eugene Garfield) to obtain detailed information (including country, institution, journal, Category, and so on) on publications in the field of TMZ; and the bibliometric software VOSviewer (VOSviewer version 1.6.17; Centre for Science and Technology Studies, Leiden University, Netherlands) to construct. The global disparities in TMZ research by country can be better visualized using the geographic heat map. Using the Geographic Heat Map add-in in a spreadsheet, a geographic heat map of TMZ articles was constructed in this paper (Microsoft Office 2021 Excel; Microsoft Corp.). The Journal Citation Reports 2020 (JCR) included each journal’s impact factors (IF) and H-indexes. Impact factor (IF) has become an internationally accepted index for journal evaluation. It is not only a measure of the usefulness and display of a journal, but also an important indicator of the academic level of a journal, and even the quality of a paper (8). The H-index is a method of evaluating academic achievement, which can accurately reflect a person’s academic achievement (9).

## Results

### General statistics

**Figure 2:**
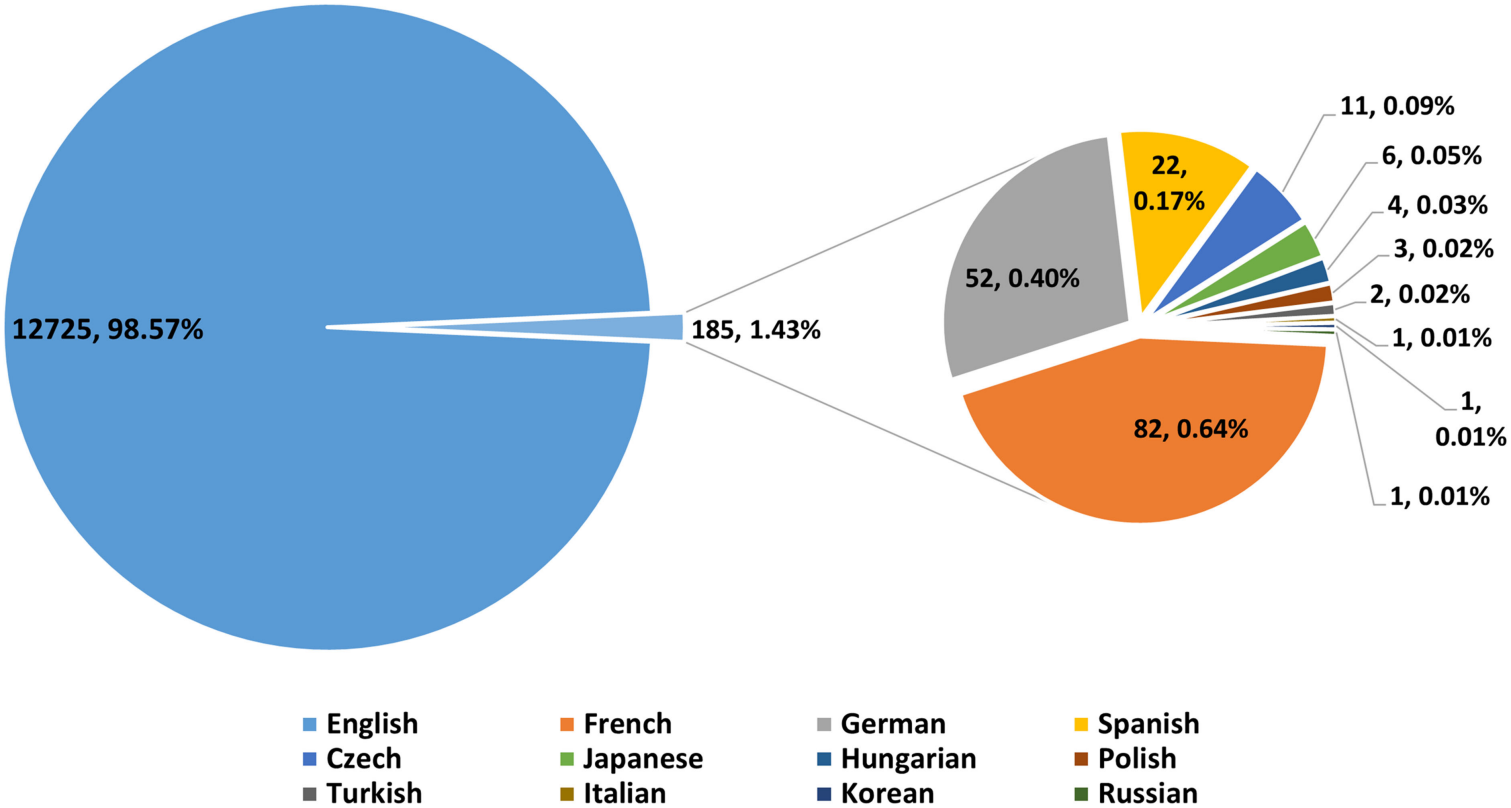
Distribution of literature languages.

From 1994 to 2021, the Web of Science (WOS) core collection database yielded 12,910 research publications on the topic of TMZ. According to the pooled study, these 12910 papers came from 104 nations, 9949 institutions, 49458 authors, 1485 journals, and 9432 grant-funded institutions. They are written in 12 languages and contain 24929 keywords. With 12,725 papers (98.57%), English-language articles are the most common, followed by French, German, and Spanish, with 82, 52, and 22 articles, respectively (**Figure 2**). The types of literature were separated into two categories: research-based literature (86.18%) and review-based literature (13.82%) (**Table 1**). This shows that the range of temozolomide-related studies mapped (countries, institutions, authors, etc.) is wide and the depth of research is deep.

**Table 1:** Distribution of literature types.

Type of Literature	Number	Percentage (%)
Research-based Literature	11126	86.18
Review-based Literature	1784	13.82

### Publication time statistics

**Figure 3:**
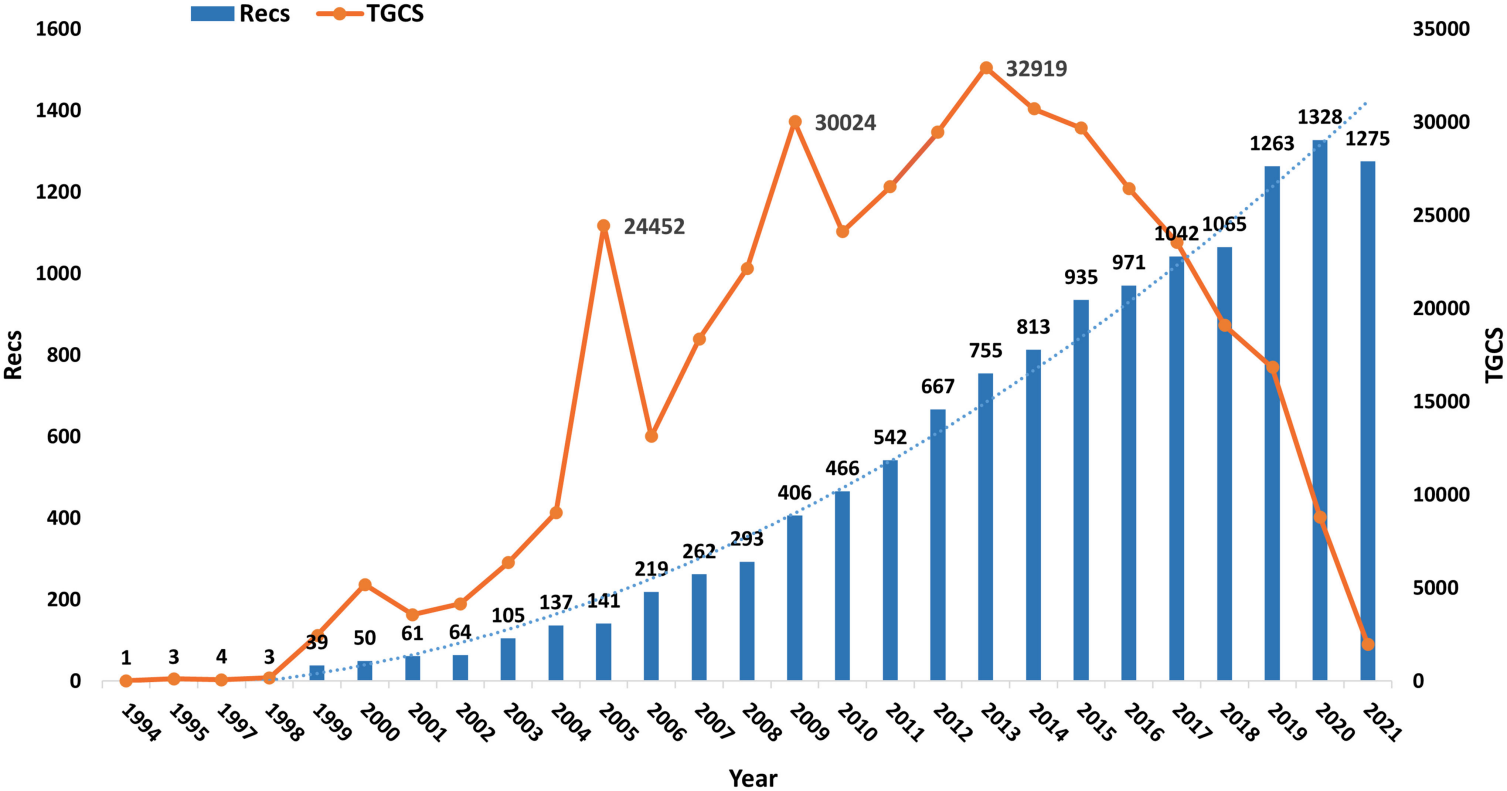
Timeline of publications and TGCS on TMZ.

The quantity of literature and its patterns reflect this topic’s current study state (10). Malcolm F. G. Stevens et al. published the first TMZ literature in 1994, indicating several different synthetic techniques employed for the overall synthesis of TMZ. This study shows the annual number of publications in the field of temozolomide about the total literature records (Recs) and global citation score (TGCS) from 1994-2021 (**Figure 3**). In terms of total literature records (Recs), the publications increased slowly from 1994 to 1998. They increased quickly from 1999 to 2021, with an average of 615 publications per year, and the maximum number was 1328 in 2020. TGCS increased rapidly from 1994 to 2015 before gradually decreasing from 2016 to 2021. The rapid growth of TGCS in 2000, 2005, and 2009 and the peak of TGCS in 2013 (highest value of 32919) indicate breakthroughs or significant discoveries in TMZ research may have occurred during these four years.

### Country characteristics

**Figure 4:**
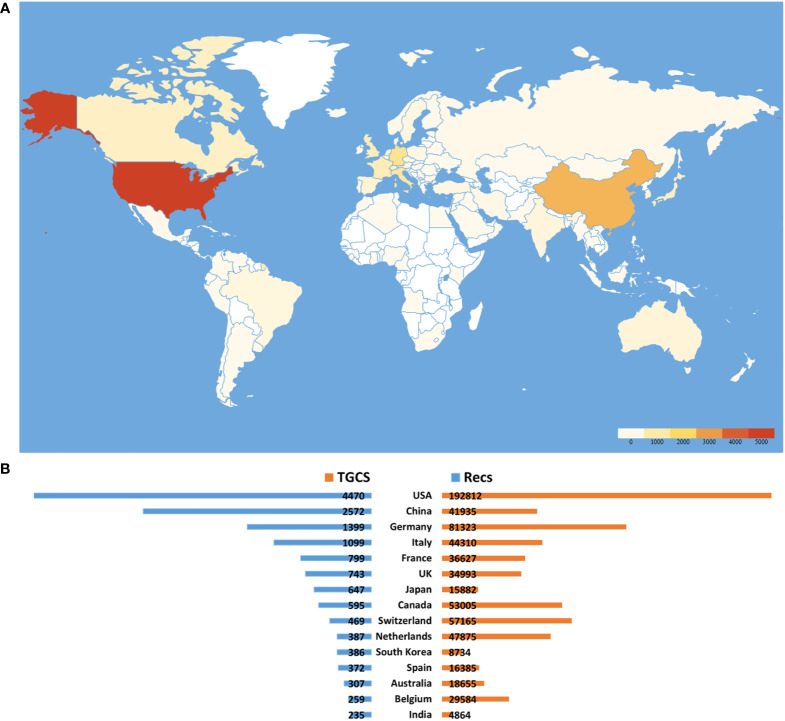
Main countries distribution on TMZ publications. **(A)** Geographical distribution of TMZ. **(B)** The top 15 most productive countries in the publication of TMZ and their corresponding TGCS.

It’s critical to examine TMZ at the country level to assess its academic impact and choose nations to focus on. Between 1994 and 2021, teams from 104 countries published articles on TMZ. **Figure 4A** depicts the global distribution of all publications, whereas **Figure 4B** depicts the 15 nations with the highest Recs and TGCSs productivity. The United States, China, Germany, and Italy were the countries with >1000 publications out of the 12910 total, with the United States having the most (4470, 34.6%), followed by China (2572, 19.9%) and Germany (1399, 10.8%). In terms of TGCS, the United States ranked first (192812, 47.1%), followed by Germany (81323, 20.1%), and the Switzerland (57165, 14.1%). In the field of TMZ, we discovered that the United States dominated both the number of publications and the overall global citation score, indicating that the United States may have the most comprehensive and in-depth research in this sector. Although China is second only to the United States in the Recs ranking, it is ranked seventh in the TGCS, indicating that China has a significant role in TMZ research. Still, its academic impact on the subject needs to be strengthened to improve its international significance.

### Academic collaboration

**Figure 5:**
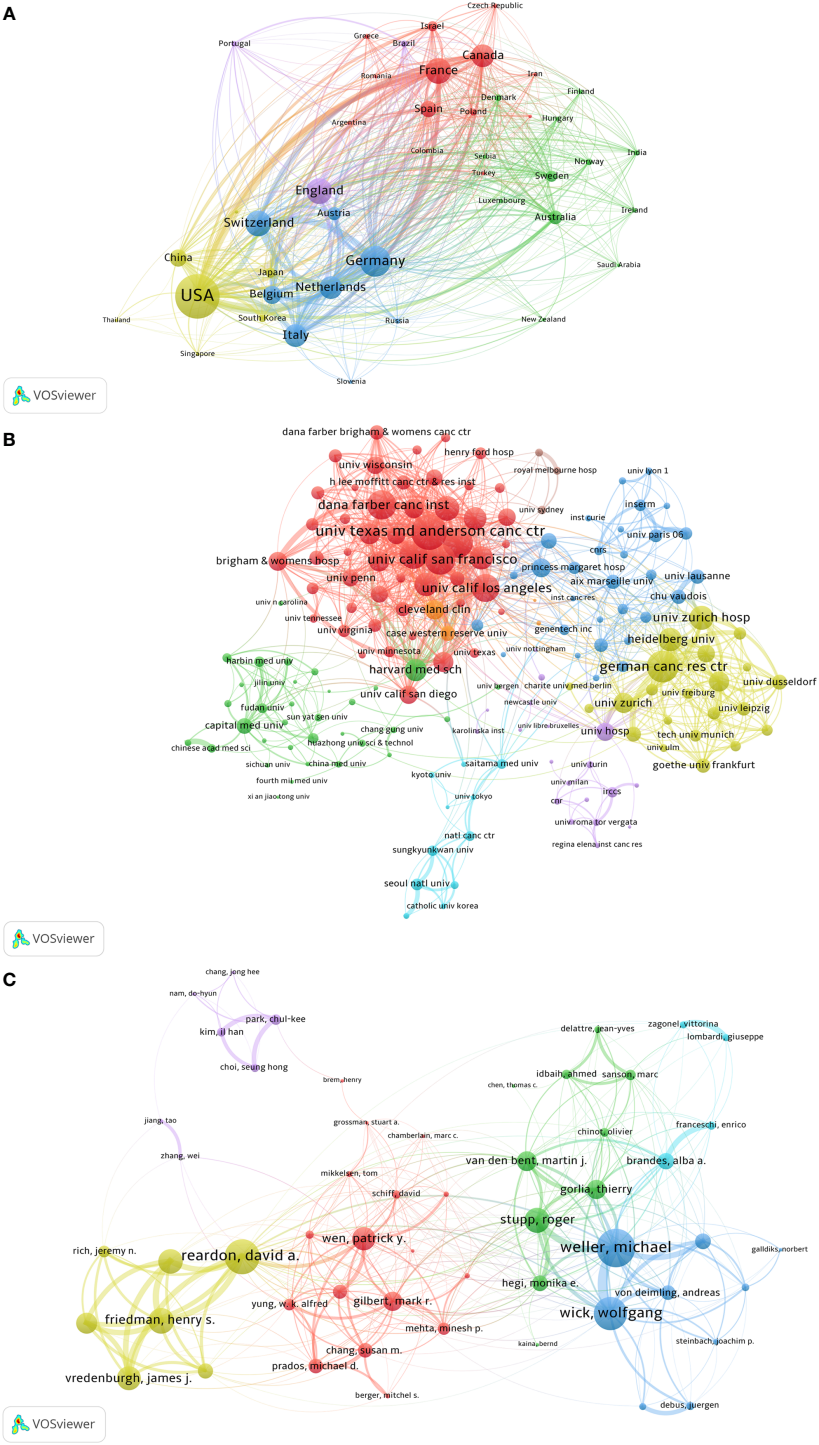
Academic collaboration between countries, institutions, and authors in the TMZ research area. **(A)** Academic collaboration between different countries. **(B)** Collaboration between different institutions. **(C)** Collaboration between different authors. From: CiteSpace, VOSviewer doi: 10.3389/fonc.2022.905868.

**Figure 6:**
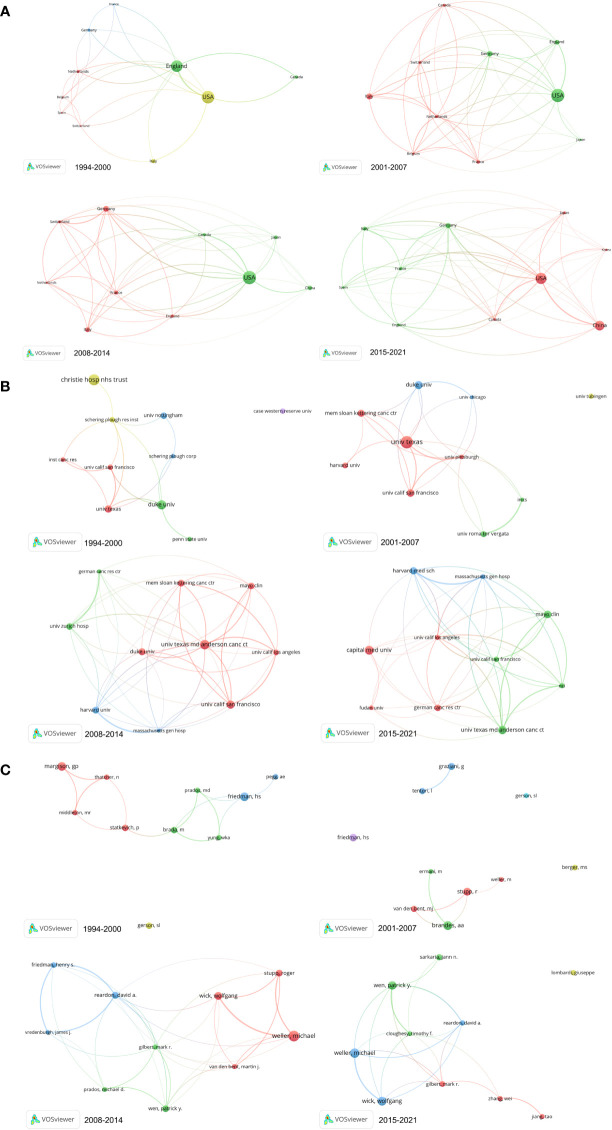
The evolution of collaborative networks among different research forces. **(A)** The evolution of collaboration networks of top 10 prolific countries. **(B)** The evolution of collaboration networks of top 10 prolific institutions. **(C)** The evolution of collaboration networks of top 10 prolific authors. From: CiteSpace, VOSviewer doi: 10.3389/fonc.2022.905868.

Academic collaboration among countries, institutions, and authors is progressively becoming the standard scientific research approach as the breadth of scientific study extends and the depth of research deepens (11). To evaluate TMZ’s academic influence and determine its research force, it’s vital to look at it from three perspectives: countries, institutions, and authors. We investigated the academic collaboration between countries, institutions, and authors in the TMZ research area(**Figure 5**) and the evolution of collaborative networks between different research forces over a period of 28 years (**Figure 6**). Each node represents a different country/institution/author. The node’s size represents the number of articles. The thickness of the connecting lines represents the power of collaboration. Each group in the collaborative network with similar attributes represents a cluster colored differently.

With a threshold of at least 25 publications, the co-occurrence analysis of countries using VOSviewer software produced a network diagram of country co-occurrence (**Figure 5A**). 51 countries have crossed the cooperation threshold. There are closer collaboration links among nations, as evidenced by the intricate overlapping linkages, and the United States, in particular, plays a crucial role in international exchange. China-USA, Germany-Switzerland, and France-Canada have the highest total collaboration intensity among the six clusters of the national cooperation network. From the evolution of the country collaboration network (**Figure 6A**), it can be observed that initially (1994–2000), the major countries researching TMZ were all located in the United States and Europe, and the closest collaboration was between the United States and the United Kingdom. Over time, Asian countries, represented by China, Japan, and Korea, joined the collaborative network of TMZ research. The United States dominated throughout the 28-year period, but in the last 7 years, China’s share of the national collaborative network has grown and has approached that of the United States. In the next 15 years, the United States may be overtaken by China.

A co-occurrence research of institutions revealed a graph of institutional co-occurrence networks with a threshold of at least 35 publications (**Figure 5B**). In **Figure 5B**, we see that 174 institutions were organized into eight distinct clusters. The University of Texas MD Anderson Cancer Center, a world-class center for cancer research, diagnosis, and treatment, was the most prolific research institution (literature = 315) and the institution with the highest collaboration intensity (total collaboration intensity = 696), according to a closer look at the top 10 collaborating institutions (**Table 2**). Second and third places, respectively, went to the German Cancer Research Center (GRC) and the University of California, San Francisco (Univ California San Francisco). In addition to the German Cancer Research Center and the University Hospital Zurich, eight of the top 10 partnering institutions are from the United States, showing that TMZ research in the United States is high. Furthermore, seven of the top ten institutions are top oncology research institutes, while the remaining three universities are among the world’s leading oncology research and treatment institutions. This shows that basic TMZ research is intimately tied to clinical applications. From the evolution of the institutional collaboration network (**Figure 6B**), it can be observed that during the period 1994-2007, the inter-institutional collaborations were not strong and the research strengths of the institutions varied widely, with Christie Hospital and University Texas being the most prominent. 2008-2014, compared to the other institutions in the institutional collaboration network The strength of the collaboration between Massachusetts General Hospital and Harvard University, German Cancer Research Center and University Zurich Hospital is outstanding compared to other institutions in the institutional collaboration network. In the last seven years, the strength of the collaboration between Massachusetts General Hospital and Harvard University was stronger than the other institutions.

**Table 2:** The top 10 most collaborative institutions.

Organization	Document	Citation	Total collaborative strength
Univ Texas MD Anderson Canc Ctr	315	18245	696
German Canc Res Ctr	203	11231	588
Univ Calif San Francisco	268	19182	553
Massachusetts Gen Hosp	175	9343	533
Dana Farber Canc Inst	156	9236	508
Univ Calif Los Angeles	201	14848	487
Mayo Clin	262	12078	482
Mem Sloan Kettering Canc Ctr	213	15737	403
Univ Zurich Hosp	125	11730	389
Univ Pittsburgh	131	8232	373

VOSviewer software was used to do a co-occurrence analysis of research authors. A threshold of at least 37 publications was employed to generate an author co-occurrence network graph, as shown in **Figure 5C**. The 50 writers are organized into six clusters, independent of the others. The strong ties that bind these six author clusters point to a global network of scholars working together. Michael Weller and Wolfgang Wick are the two nodes in the collaborative network with the most prominent publication volume and collaboration intensity. The core nodes of the six collaborative network clusters are Weller Michael, Reardon David A., Stupp Roger, Wen Patrick Y., Brandes Alba A., and Park chuckle. From the evolution of the author collaboration network (**Figure 6C**), it can be observed that in the first 14 years (1994–2007), the inter-institutional collaborations were in a relatively simple stage of development and did not form mature networks, but only chains of collaboration. In the last 14 years, institutional collaborations have been further developed. between 2008-2014, compared to other authors in the author collaborative network, Weller Michael-Stupp Roger-Wick Wolfgang and Reardon David A.-Friedman Henry S.- Vredenburgh James J. The two author clusters are the most prominent in the author collaboration network. In the last 7 years, two author groups, Weller Michael-Wick Wolfgang and Wen Patrick Y.-Cloughesy Timothy F., have replaced other authors as the core author groups in the author collaborative network.

### Funding agency distribution

**Figure 7:**
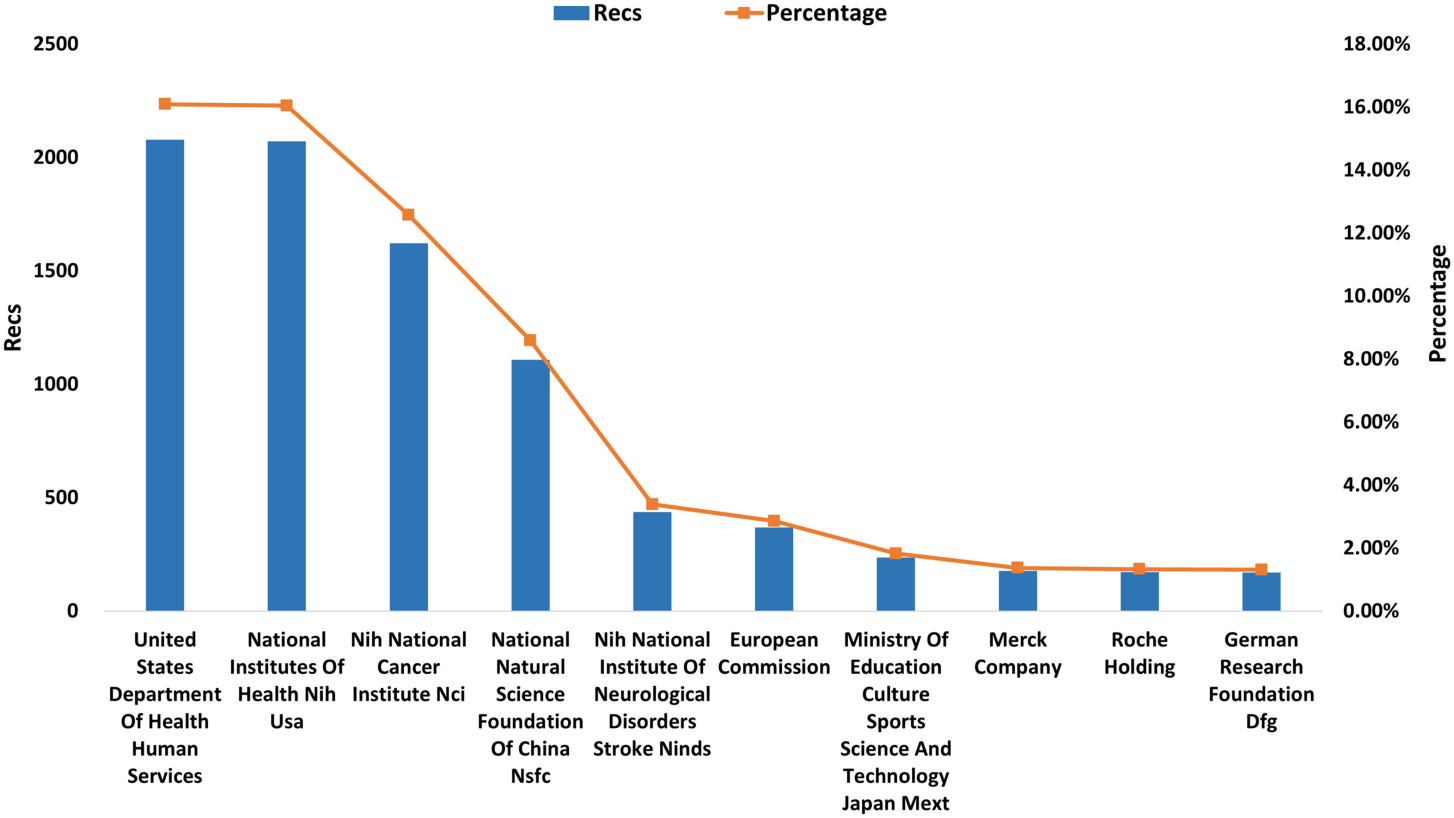
The top 10 productive funding agencies in the TMZ area.

The research direction and content in TMZ are reflected in the distribution of funding agencies (12). The top ten institutions that funded TMZ-related research the most from 1994 to 2021, accounting for 65.41% of the total number of publications (**Figure 7**). The top four funding agencies, including the USA Department of Health and Human Services, the National Institutes of Health (NIH) USA, the Nih National Cancer Institute (NCI), and the National Natural Science Foundation of China (NSFC), with 6,882 articles funded, accounting for more than half (53.31%) of the total number of publications. The top three research funding agencies are all from the United States, accounting for 44.72% of all publications, consistent with the high number of publications, high citations, and high level of collaboration, reflecting the United States’ leading position in the field of TMZ research. The National Natural Science Foundation of China (NSFC) ranks fourth behind the three USA research funding organizations, with 1109 publications financed, demonstrating China’s contribution to TMZ research. Two pharmaceutical corporations (Merck Company and Roche Holding)are in the top ten research funding organizations, indicating that basic and clinical TMZ research are closely linked.

### Institution and author contributions

**Figure 8:**
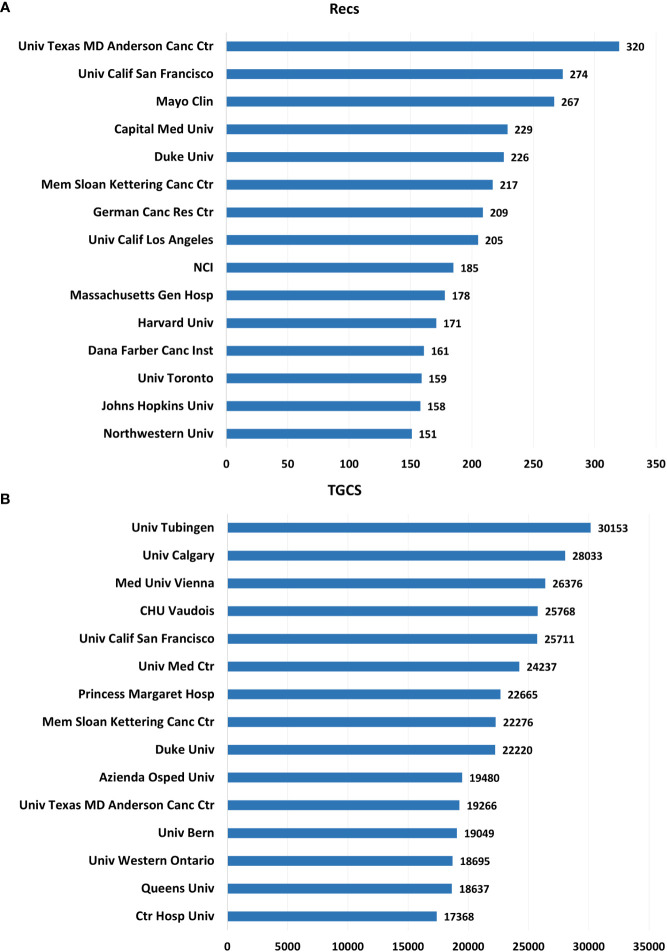
**(A)** The top 15 productive institutions in TMZ by Recs. **(B)** The top 15 productive institutions in TMZ by TGCS.

Authors and research institutions are vital forces driving academic development and innovation in a particular field, so this study is important to analyze the main institutions and essential authors in TMZ (13). This study presents the top 15 productive institutions in the Recs and TGCS (**Figure 8**). According to Recs and TGCS, the 15 research institutions can be divided into three categories: a) Institutions represented by University Texas MD Anderson Cancer Center, University Calif San Francisco, Duke University, and Mem Sloan Kettering Cancer Center are characterized by high TGCS and high Recs of publications. b) Institutions represented by University Tubingen, University Calgary, and the Medical University of Vienna are characterized by high TGCS and low Recs of publications. c) Institutions represented by Mayo Clinic, Capital Medical University, and The institutions represented by Mayo Clinic, Capital Medical University, and German Cancer Research Center are characterized by low TGCS and high Recs of publications.

**Figure 9:**
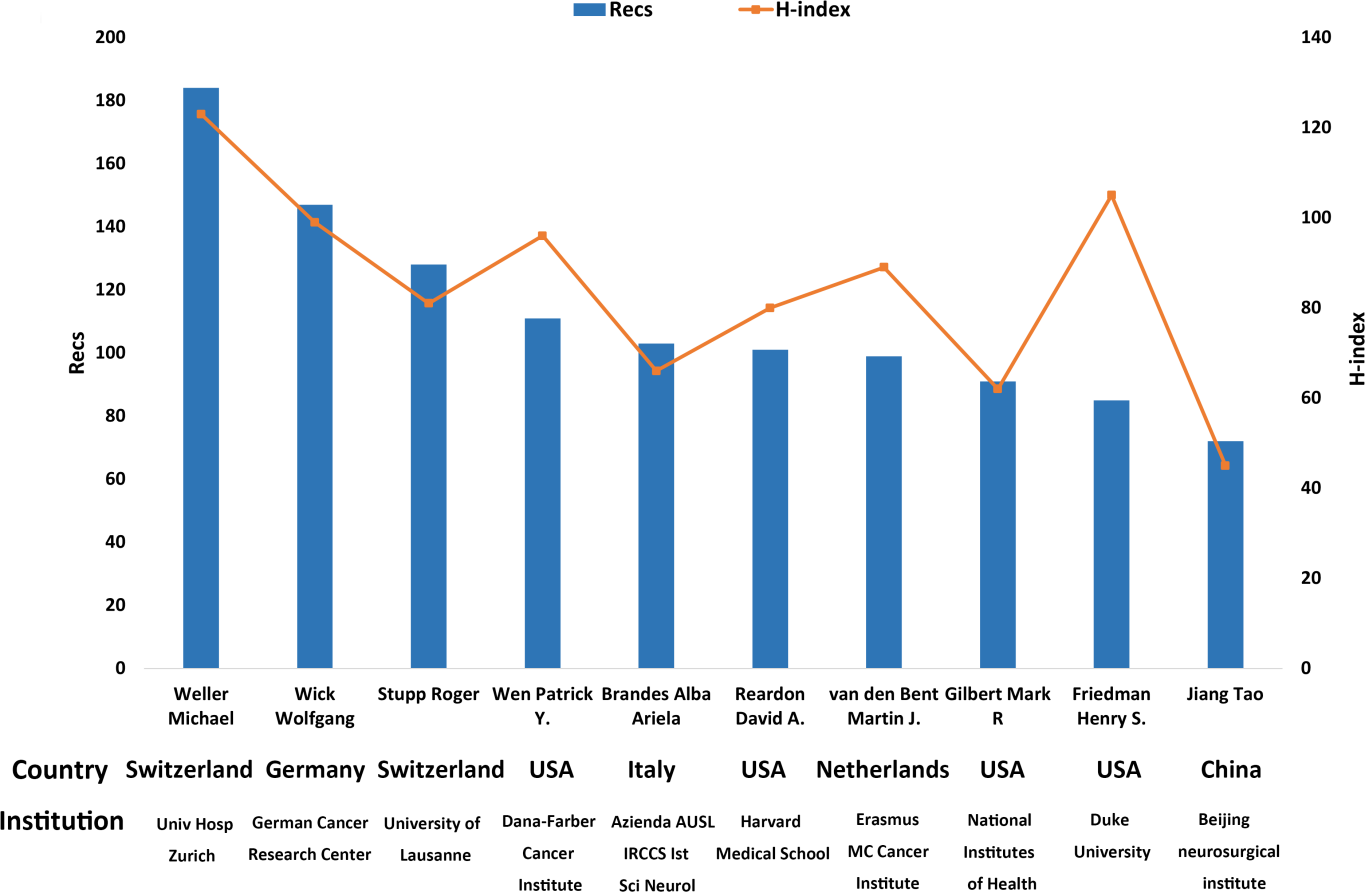
The top 10 most productive authors in the TMZ research area.

All authors are rated according to their number of publications and h-index in this study to investigate the most influential specialists in TMZ research for 1994-2021 (**Figure 9**). The H-index predicts future scientific accomplishment outperforms other measures (total citation count, citations per paper, and total paper count) (9). Weller Michael of the University Hospital Zurich is not only the most influential author (Recs=184) but also the author with the highest h-index (H-index=123), multiple highly referenced works, and the most significant overall TGCS score (TGCS=50816). With an h-index of 105 and a Recs rating of 85, Friedman Henry S. of Duke University, USA, has the second-highest h-index. Tao Jiang of the Beijing Neurosurgical Institute, the sole Chinese among the top ten specialists, has made significant contributions, with 72 and h-index of 45, respectively.

### Journal performance

The research of journals identifies the specific directions and disciplines involved in a field and identifies the critical journals in that field, which provide a reference for researchers (14). Impact factor (IF) is a key indicator of the academic level of journals and even the quality of papers and a measure of their utility and display (8), which was proposed by Eugene Garfield in 1972 (15). This research lists the details of the journals involved in the top ten TMZ publications in terms of the number of publications (**Table 3**), six of which are from the United States, two from Switzerland, and one from the United Kingdom and Greece. It is worth noting that the top ten journals in terms of the number of publications account for 22.2% of the total number of publications, which indicates that the selected journals are representative and of research significance. The Journal of Neuro-Oncology has the most publications (Recs=879), but it also has the highest TGCS score (TGCS=23102). Seven of the top ten journals in terms of articles are dedicated to oncology research. In contrast, two are dedicated to neuro-oncology, reflecting the focus of TMZ research in cranial cancers.

**Table 3:** The top 10 journals ranked by their Recs in the TMZ area.

Journal	Country	Recs	TLCS	TGCS	IF(2020)	H-Index	ISSN
Journal Of Neuro-Oncology	United States	879	7851	23102	4.130	105	0167-594X
Neuro-Oncology	United States	432	6952	22493	12.300	105	1522-8517
Oncotarget	United States	256	1527	7405	0	91	1949-2553
Clinical Cancer Research	United States	253	6715	20173	12.531	292	1078-0432
Plos One	United States	250	0	7345	3.240	268	1932-6203
Cancers	Switzerland	171	26	1220	6.639	53	2072-6694
Scientific Reports	England	171	0	2629	4.379	149	2045-2322
Frontiers In Oncology	Switzerland	166	0	1304	6.244	60	2234-943X
Anticancer Research	Greece	149	700	2280	2.480	110	0250-7005
World Neurosurgery	United States	142	370	1469	2.104	85	1878-8750

### Category analysis

**Figure 10:**
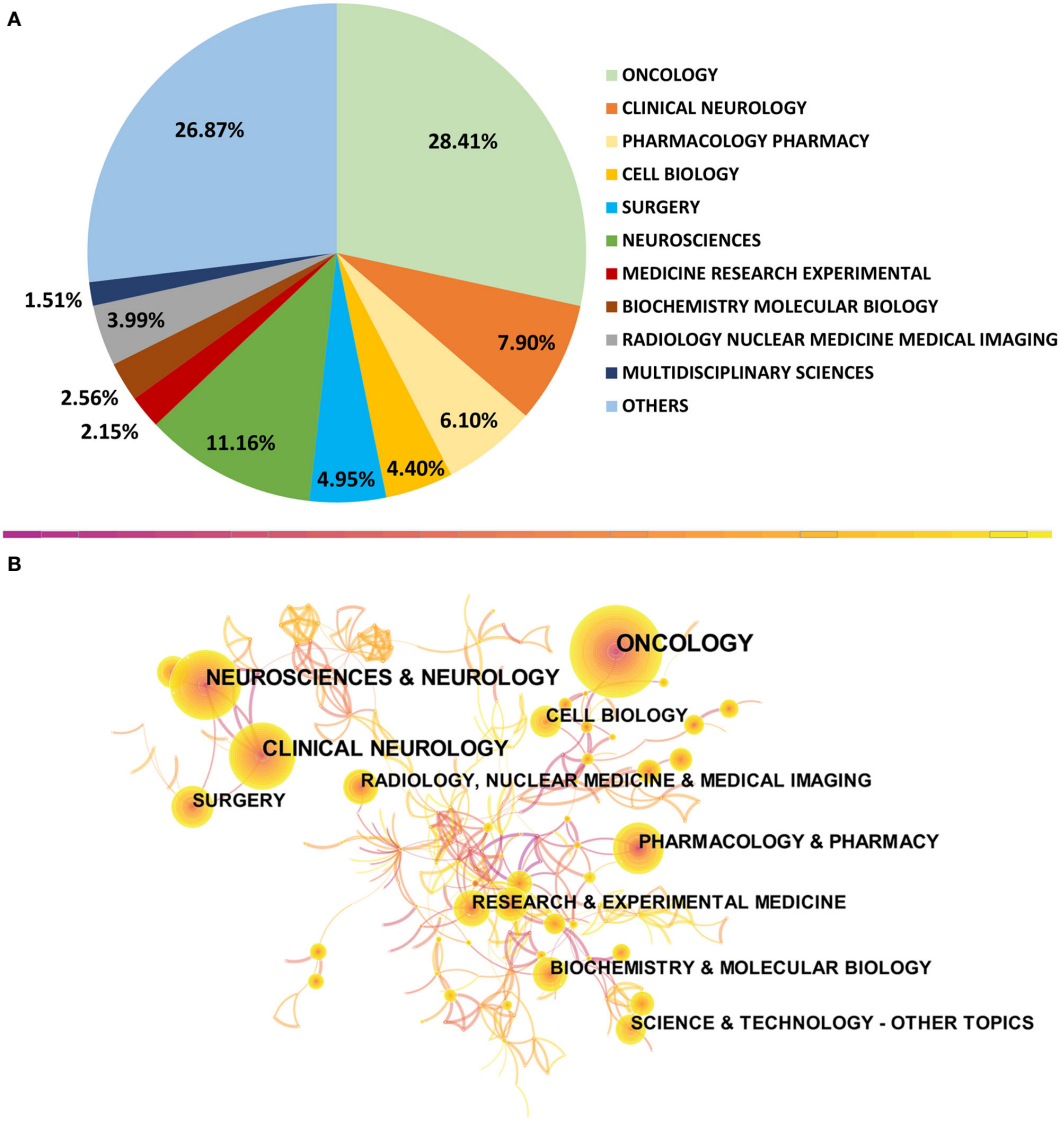
Analysis of categories. **(A)** The top 10 most published subjects within the TMZ area. **(B)** A category visualization of TMZ research. Each node represents a category, with the node’s size representing the frequency of occurrence and the color shade representing the year’s distance. The connecting line’s thickness between the nodes represents the strength of their interactions, while the connecting lines between the nodes represent their interrelationships.

Research on the categories involved in a field can identify the field’s development direction and research hotspots (16). This study conducted a sectoral percentage analysis (**Figure 10A**) and category co-occurrence chart analysis (**Figure 10B**) for the 10 most involved topics in the TMZ field.

According to **Figure 10A**, Oncology (3668 records, 28.41%), clinical neurology (3469 recordings, 26.87%), and pharmacology/pharmacy (1441 records, 11.16%) make up the top three categories, accounting for 66.44% of all articles. In addition, the bottom ten research categories, such as cell biology, surgery, and neurosciences, may be developing research areas and thus equally informative.

**Figure 10B** depicts the visual analysis of the TMZ research categories from 1994 to 2021. The 10 most cited articles’ categories were reviewed to find more than 512 times cited. According to the Co-occurrence network, the most crucial research categories in TMZ are oncology, clinical neurology, and pharmacology/pharmacy. In contrast, biochemistry/molecular biology, radiology, nuclear medicine/medical imaging have a high citation frequency and a newer year of cited literature, indicating a possible emerging research hotspot.

### Keyword detection and burst analysis

Keywords can express the primary content of a piece of writing. High-frequency keywords can reveal new research trends, whereas emergent keywords can reveal new research hotspots (17). After de-duplication and synonyms, 24928 keywords were extracted from 12910 texts. With 118 as the minimum number of keyword occurrences, 176 were eventually chosen, and the co-occurrence cluster analysis yielded three clusters.

**Figure 11:**
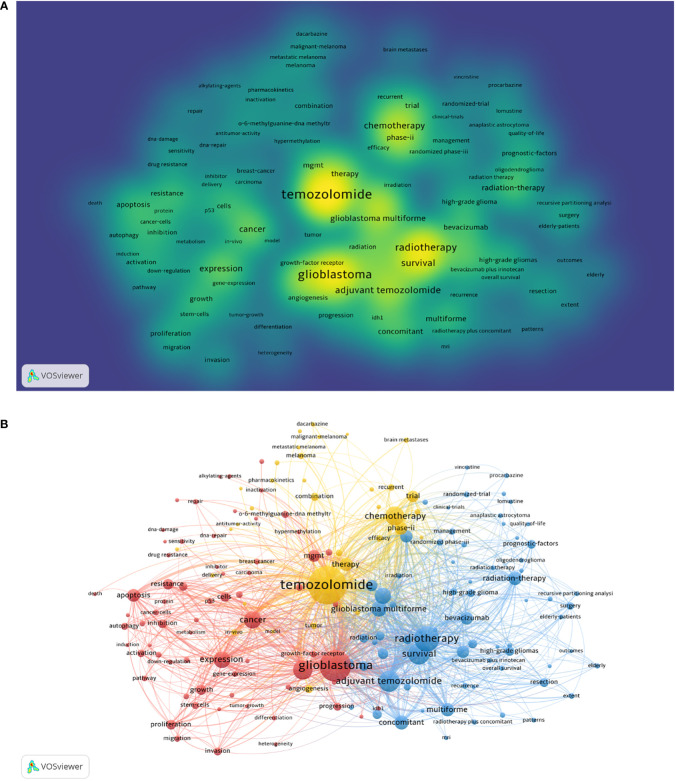
Keywords analysis. **(A)** Heatmap visualization analysis of keywords appearing more than 118 times in TMZ field from 1994-2021. Warmer spots indicate hotter research keywords, and cooler spots show colder keywords. **(B)** Cluster analysis of keywords appearing more than 118 times in TMZ field, 1994-2021. From: CiteSpace, VOSviewer doi: 10.3389/fonc.2022.905868.

The top ten most frequent keywords were TMZ (7434), glioblastoma (4873), radiotherapy (2966), survival (2266), adjuvant TMZ (2247), glioma (2095), chemotherapy (1928), cancer (1831), expression (1697), and malignant glioma (1398)(**Figure 11A**). Each node in **Figure 11B** represents one of the 176 keywords, with the node’s size indicating the frequency of occurrence. The number of times two keywords appear simultaneously is represented by the connecting line between the nodes, and its thickness denotes the frequency of appearance. The three clusters represented by different colors are as follows: 1) Mechanism in red: glioblastoma, expression, MGMT, proliferation, and o-6-methylguanine-DNA methyltransferase; 2) Clinic treatment in yellow: TMZ, chemotherapy, therapy, immunotherapy, and combination; 3) Drug design in blue: radiotherapy, adjuvant TMZ, bevacizumab, and survival.

**Figure 12:**
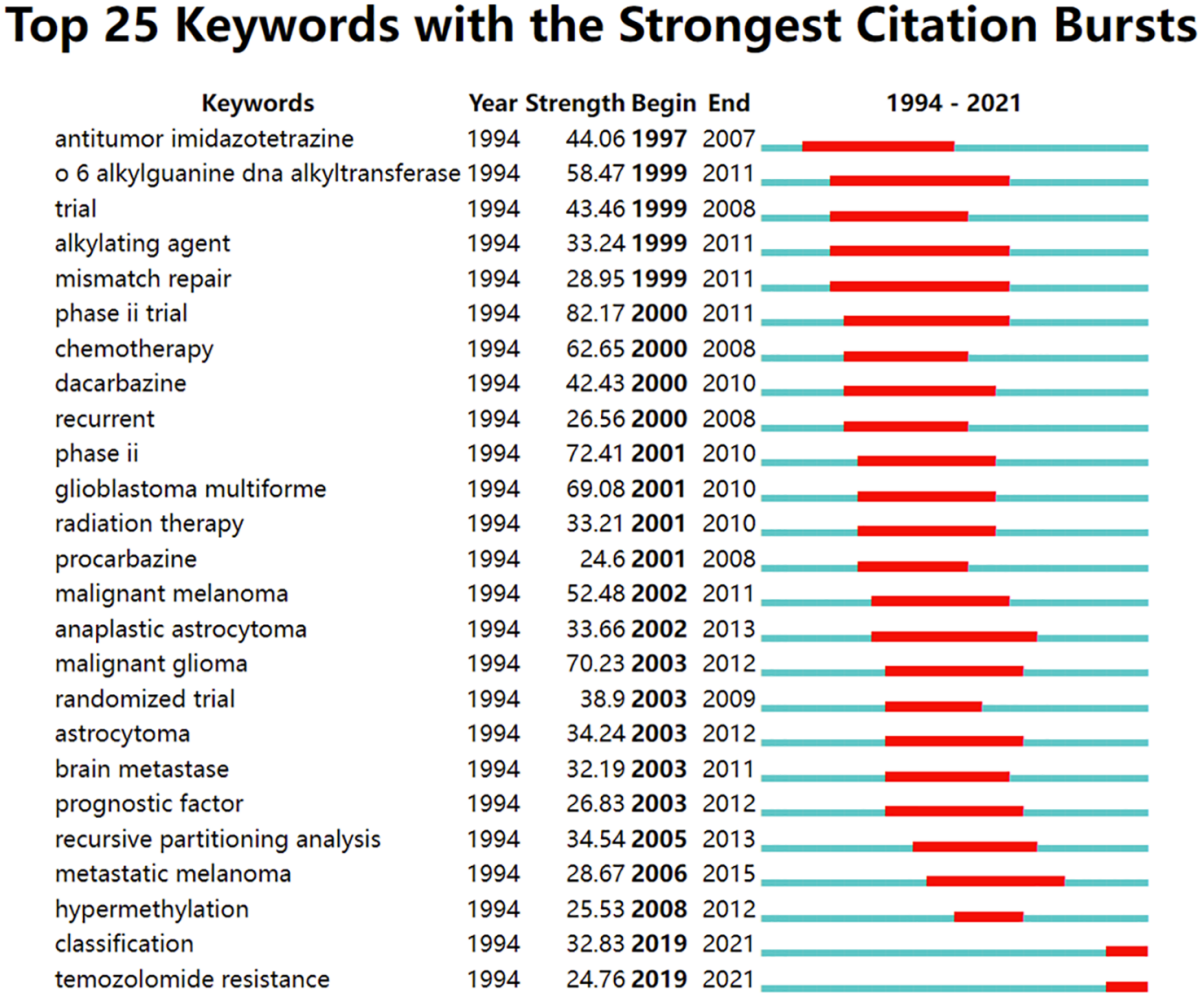
The burst detection of keywords of TMZ area in chronological order, 1994-2021.

In addition, we have analyzed the burst keywords (**Figure 12**). The results showed that burst keywords lasting more than 5 years are: antitumor imidazole-tetrazine, O6-alkylguanine-DNA alkyltransferase, trial, alkylating agent, mismatch repair, phase II trial, chemotherapy, dacarbazine, recurrent, glioblastoma multiforme, radiation therapy, procarbazine, malignant melanoma, anaplastic astrocytoma, malignant glioma, randomized trial, brain metastase, prognostic factor, recursive partitioning analysis and emergent terms that persist to the present are classification, TMZ resistance.

### Co-citation analysis and roadmap of TMZ development

**Figure 13:**
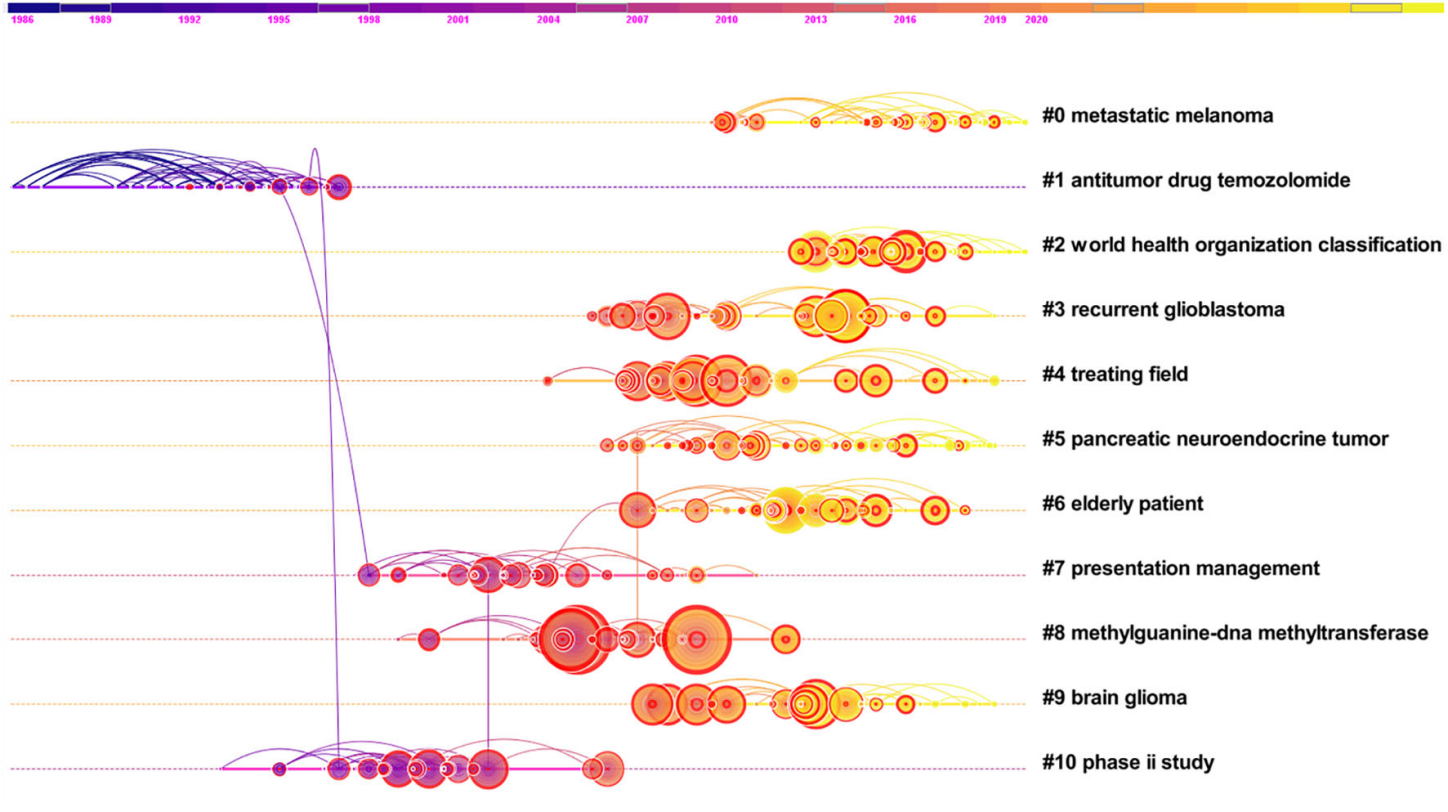
Visualized timeline of co-citation clusters in the TMZ research area.

A co-citation relationship is defined as two or more papers simultaneously cited by one or more later articles. The core literature that plays a crucial role in the evolution of claims in a subject is displayed in literature co-citation analysis and the shifting trends in research focus. In this research, we do a co-citation analysis of 12,910 documents, setting the time slice length to one year, the period to 1994-2021, showing the number of clusters to 11 (clusters are listed in numerical order by tag), and creating a timeline diagram to show the significant clusters of co-citations of references (**Figure 13**). The parameters of this timeline graph are N = 721, E = 778, totaling 721 nodes and 778 linkages; The network’s density is 0.003, and its modularity is Q = 0.9214. The degree of modularity Q reflects the network’s modularity, and modularity Q of 0.4 to 1 usually satisfies or approaches the clustering requirements (18). Thus the network meets the standard requirements for clustering. The silhouette value is used to quantify the homogeneity of the network, and a silhouette value greater than 0.7 is considered a convincing clustering. Therefore, all of our clusters meet this criterion(**Table 4**). The timeline diagram shows 139 nodes, and the lines connecting these nodes represent co-referential relationships. Larger nodes reflect more frequent citations, while the lighter nodes/lines represent later publication dates along the dotted lines connecting each cluster label. The red circles in the nodes indicate sudden co-citations, alluding to a spike in the frequency of mentions of a publication at a particular time, indicating a research trend on the subject.

**Table 4:** The detailed information of the 11 clusters in Figure.

Cluster-ID	Cluster label	Size	Silhouette	Mean(Year)
0	Metastatic Melanoma	44	1	2015
2	World Health Organization Classification	33	1	2016
3	Recurrent Glioblastoma	33	1	2011
4	Treating Field	33	0.987	2010
5	Pancreatic Neuroendocrine Tumor	33	1	2012
1	Antitumor Drug TMZ	30	0.979	1993
6	Elderly Patient	30	1	2012
8	Methylguanine-DNA Methyltransferase	30	1	2006
7	Presentation Management	28	0.949	2003
10	Phase II Study	27	1	1999
9	Brain Glioma	26	0.986	2013

Based on the timeline diagram, we can obtain the main popular themes of TMZ research during the period 1994-2021. The evolution of the main research themes during these 28 years is as follows, from 1987 to 1997, cluster #1 antitumor drug TMZ received attention from researchers as the earliest hot topic in TMZ research. There was overlap between the different research hotspots in the subsequent period. From 1996 to 2012, cluster #10 phase II study, cluster #7 presentation management, and cluster #8 methylguanine-DNA methyltranferase emerged sequentially as major popular themes, with cluster #8 having a high concentration of nodes and citation bursts in the timeline graph and being an important theme in TMZ research. cluster #0 metastatic melanoma, cluster #2 world health organization classification, cluster #3 recurrent glioblastoma, cluster #4 treating field, cluster #5 pancreatic neuroendocrine tumor, cluster #6 elderly patient, cluster #9 brain glioma are the hot topics of TMZ research from 2005 to 2020.

In addition, we collect the first 15 articles of the TGCS, as shown in **Table 5**, to gain a more thorough understanding of critical events in the history of the TMZ sector. These publications cover several pivotal moments in the development of TMZ. The FDA approved TMZ capsules in 1999 for treating adult patients with refractory mesenchymal astrocytoma, and by the EU in the same year for the treatment of glioblastoma multiforme that had progressed or recurred after standard therapy. Following more rigorous drug toxicity and efficacy studies, TMZ was widely used as an effective first-line chemotherapeutic agent for treating patients with GBM (19). TMZ was proven effective as dacarbazine as an oral option for individuals with advanced metastatic melanoma by Middleton, MR et al. in 2000 (20). Hegi, ME, et al. discovered in 2005 that patients with glioblastoma who had a methylated MGMT (O 6-methylguanine -DNA methyltransferase) promoter benefited from TMZ. Still, those who did not have a methylated MGMT promoter did not (21). The addition of TMZ to radiation for newly diagnosed glioblastoma resulted in clinically relevant, and statistically, significant survival increases with minimal additional damage, according to Stupp, R. et al. in 2005 (22). This treatment regimen for glioblastoma has now become the gold standard. Gentoo Liu et al. discovered in 2006 that CD133-positive tumor stem cells were highly resistant to chemotherapy treatments (led by TMZ). CD133-positive cells with high expression of BCRP1 and MGMT and anti-apoptotic proteins and inhibitors of the apoptotic protein family may be linked to this resistance (23). In conjunction with irinotecan, Bevacizumab effectively treated recurrent glioblastoma multiforme with mild toxicity by Vredenburgh, James J., et al. in 2007 (24). This study paved the way for bevacizumab to be added to the future treatment options for recurrent glioblastoma. Chin, L. et al. discovered common mutations in the phosphatidylinositol-3-OH kinase regulatory subunit genePIK3R1 in 2008 and provided a network perspective of the pathways affected in glioblastoma growth (25). In a randomized phase III study published in 2009, Stupp, Roger discovered that glioblastoma patients treated with TMZ and radiation improved median and 2-year survival rates. MGMT promoter methylation was the strongest predictor of outcome and benefit of TMZ chemotherapy (26). Two studies published in 2014 by Chinot, Olivier L., et al., and Gilbert, Mark R., et al., found that using bevacizumab as a first-line treatment did not enhance overall survival in patients with newly diagnosed glioblastoma. Although progression-free survival was extended, the anticipated goal of improvement was not met. This shows that bevacizumab isn’t a perfect replacement for TMZ as the recommended glioblastoma treatment (27, 28). Ribas, Antoni, and colleagues established pembrolizumab as a new standard of care for ipilimumab-refractory melanoma in 2015 (29).

**Table 5:** The top 15 most cited articles.

Authors	Journal	IF(2020)	H-Index	Title	TGCS	TLCS
Stupp R et al.	New Engl J Med	91.245	933	Radiotherapy plus concomitant and adjuvant TMZ for glioblastoma	12496	5994
Chin L et al.	Nature	49.962	1096	Comprehensive genomic characterization defines human glioblastoma genes and core pathways	5061	702
Stupp R et al.	Lancet Oncol	41.316	274	Effects of radiotherapy with concomitant and adjuvant TMZ versus radiotherapy alone on survival in glioblastoma in a randomized phase III study: 5-year analysis of the EORTC-NCIC trial	4700	2702
Hegi ME et al.	New Engl J Med	91.245	933	MGMT gene silencing and benefit from TMZ in glioblastoma	4462	2502
Friedman HS et al.	J Clin Oncol	44.544	494	Bevacizumab Alone and in Combination With Irinotecan in Recurrent Glioblastoma	1683	641
Gilbert MR et al.	New Engl J Med	91.245	933	A Randomized Trial of Bevacizumab for Newly Diagnosed Glioblastoma	1494	651
Chinot OL et al.	New Engl J Med	91.245	933	Bevacizumab plus Radiotherapy-TMZ for Newly Diagnosed Glioblastoma	1400	706
Chen J et al.	Nature	49.962	1096	A restricted cell population propagates glioblastoma growth after chemotherapy	1350	221
Liu GT et al.	Mol Cancer	27.401	103	Analysis of gene expression and chemoresistance of CDI33(+) cancer stem cells in glioblastoma	1313	0
Omuro A et al.	Jama-J Am Med Assoc	56.272	622	Glioblastoma and Other Malignant Gliomas A Clinical Review	1221	268
Vredenburgh JJ et al.	J Clin Oncol	44.544	494	Bevacizumab plus irinotecan in recurrent glioblastoma multiforme	1074	425
Ribas A et al.	Lancet Oncol	41.316	274	Pembrolizumab versus investigator-choice chemotherapy for ipilimumab-refractory melanoma (KEYNOTE-002): a randomized, controlled, phase 2 trial	1038	5
Van Meir EG et al.	Ca-Cancer J Clin	508.702	144	Exciting New Advances in Neuro-Oncology The Avenue to a Cure for Malignant Glioma	972	1
Middleton MR et al.	J Clin Oncol	44.544	494	Randomized phase III study of TMZ versus dacarbazine in the treatment of patients with advanced metastatic malignant melanoma	908	304
Sanai N et al.	J Neurosurg	5.115	189	An extent of resection threshold for newly diagnosed glioblastomas	868	214

### Emerging new research frontiers

The concentration and difficulty of scientific study are on research frontiers (30). The government, institutions, and researchers benefit from a current and accurate understanding of research frontiers. Because new papers take time to gain traction, this study examined articles published in the last five years (2017–2021) in eight TMZ-related journals with high impact factors and H-indexes and ranked publications in these journals by TGCS, selecting those with high TGCS, resulting in 35 articles. The eight selected journals include CA-A Cancer Journal for Clinicians (IF=508.702,H-index=144), New England Journal of Medicine (IF=91.245,H-index=933), JAMA- Journal of The American Medical Association (IF=56.272,H-index=622), Nature (IF=49.962,H-index=1096), The Lancet Oncology (IF=41.316,H- index=274), Science (IF=47.728,H-index=1058), Molecular Cancer (IF=27.401,H-index=103), Journal of Clinical Oncology (IF=44.544,H- index=494).

These 35 publications were grouped into the five categories below (with some overlap between them) and were partially compatible with the previous category, co-citation, and keyword analyses. 1) Immunotherapy for cancer (31–34). In recent years, tumor immunotherapy has developed rapidly, and FDA has approved several for clinical application. Immunotherapy against glioblastoma occupies a significant portion of the TMZ literature. EGFRvIII-positive glioblastoma patients were treated with Rindopepimut (a vaccination targeting the EGFR deletion mutation EGFRvIII) in addition to standard chemotherapy in 2017 randomized, double-blind, phase III trial (ClinicalTrials.gov, NCT01480479). Rindopepimut did not improve survival in patients with newly diagnosed glioblastoma, according to the findings (31). To demonstrate the effect of immunotherapy in glioblastoma, combination medications, including Rindopepimut, may be required. 2) Resistant to drugs (35–39). Acquired drug resistance is a major challenge in the clinical treatment of glioblastoma, and long noncoding RNAs have been shown to play a role in chemotherapy resistance. In 2020, researchers concluded from ex vivo experiments that SNHG12 could serve as a promising therapeutic target to surmount TMZ resistance, thereby improving the clinical efficacy of TMZ chemotherapy (35). 3) Tumor-treatment fields (40–42). Tumor electric field therapy is a novel oncology treatment technique that has shown significant prognostic results in combination with temozolomide to treat neoplastic glioblastoma and is now included in cancer treatment protocols in several countries. In 2017, researchers discovered that adding maintenance TMZ chemotherapy to Tumor-treating fields resulted in statistically significant improvements in progression-free survival and overall survival compared to maintenance TMZ alone in a randomized clinical trial in patients with glioblastoma (40). 4) Trials of novel anticancer drug combinations (32, 43–45). The emergence of TMZ resistance has increased the need for novel anticancer drugs. As a result, there is an increasing number of clinical studies on the combination of antitumor drugs today. In a randomized European phase II experiment published in 2021 (ClinicalTrials.gov, NCT01355445), researchers discovered that adding TMZ to Vincristine-Irinotecan increased chemotherapeutic efficacy in patients with recurrent rhabdomyosarcoma while increasing toxicity to an acceptable level (43). 5) Studies involving older and young patients (46–49). The specificity research of elderly cancer patients is different from that of young children cancer patients and is of great significance. Researchers discovered that adding bevacizumab to radiation therapy + TMZ did not benefit newly diagnosed high-grade gliomas in a randomized, parallel-group, multicenter trial published in 2017 (ClinicalTrials.gov identifier: NCT01390948) (47). Researchers determined in 2018 that short-term radiation therapy combined with TMZ resulted in longer life in older individuals with glioblastoma than a short course of radiation therapy alone (ClinicalTrials.gov number, NCT00482677) (46).

## Discussion

We used several visual analysis applications to undertake a bibliometric study of 12,910 TMZ papers in the Web of Science core database from 1994 to 2021.

The number of TMZ publications has increased annually between 1994 and 2021, demonstrating the excellent growth rate and promising future of the field. In terms of the country, the United States holds a commanding lead, with the most significant number of publications and the best academic reputation. This is attributed to the help of major medical research institutions such as the University of Texas M. D. Anderson Cancer Center, the University of California, San Francisco, Memorial Sloan-Kettering Cancer Center, and the Mayo Clinic. It’s worth noting that the University of Texas MD Anderson Cancer Center in the United States ranks first in both Recs and TGCS among all research institutes, indicating that this institution has had a significant impact on TMZ research. Among the top ten most influential authors, Wen Patrick Y., Reardon David A., Gilbert Mark R., and Friedman Henry S., are also linked with USA research institutes. Other countries, such as China, Germany, Italy, and France, are similarly crucial research forces, albeit with slightly different Recs and TGCS than the United States. China has recently rated second only to the United States in terms of publications but seventh in terms of TGCS, indicating that China’s academic influence in this sector needs to be reinforced to boost its global impact. The United States leads by a wide margin in several industries in terms of academic collaborations. Among research institutions, the University of Texas MD Anderson Cancer Center, the German Cancer Research Center, and the University of California, San Francisco had the most; among authors, Weller Michael had the collaborative network with the highest total intensity of collaboration. The top three most influential writers, according to the author h-index, are Michael Weller from University Hospital Zurich in Switzerland, Henry S. Friedman from Duke University in the United States, and Wolfgang Wick from German Cancer Research Center in Germany. According to the author’s collaboration network, the most active collaboration between Weller Michael and Wick Wolfgang has also taken place, maximizing their strengths and strengthening their academic influence.

The results of the annual co-citation timeline chart, the historical evolution of TMZ, and the research hotspots in different periods are analyzed. The discovery of TMZ (50), which has excellent anti-cancer activity, by Stevens’ group at Aston University in the UK, funded by the charity Cancer Research Campaign (CRC) in 1987, was the beginning of TMZ research. This led to the rapid development of research on TMZ in the fields of drug synthesis and oncology, and gradually began to spread to other fields such as neurology and clinical surgery. TMZ was transferred to Schering-Plough for development and was approved by the FDA (51) and the EU (2) in 1999 for the treatment of recurrent glioblastoma in adults, and began to be used in medical institutions under the label of an anti-tumor drug. This led to a global spread of TMZ research efforts, with the number of countries studying TMZ spreading from the United Kingdom and the United States at the beginning to other countries around the world; the number of scientists working closely together on TMZ, represented by Weller Michael of the University Hospital of Zurich in Switzerland and Wick Wolfgang of the German Cancer Research Center, began to increase;TMZ’s research disciplines began to spread to molecular biology, cell biology, radiology, immunology and other disciplines.After numerous clinical trial studies (52, 53) and cancer management studies (54, 55), it was found that MGMT (O6-methylguanine-DNA-methyltransferase), a DNA damage repair protein, was a key factor in TMZ chemoresistance. It was shown that overexpression of MGMT and promoter methylation made tumor cells more effective in resisting TMZ drug toxicity, suggesting a close relationship between MGMT and tumor drug chemoresistance and a direct relationship with patient prognosis (21, 56–58). TMZ clinical treatment of oncological diseases (metastatic melanoma (59), recurrent glioblastoma (60), pancreatic neuroendocrine tumor (61), brain glioma (62)), the clinical trials in elderly oncology patients (48, 49), and the World Health Organization classification of TMZ-treated diseases (63) became popular topics for TMZ between 2007 and 2020. The above analysis is consistent with the statistical results of TMZ keywords and TMZ study categories, reflecting in general that the current research hotspot of TMZ is how to better utilize TMZ (with overcoming drug resistance of TMZ as the main challenge) to better treat cancer (mainly glioma). In terms of future research directions, combining the results of this study we can conclude that the diagnosis and treatment of cancer (mainly glioma, supplemented by melanoma, refractory rhabdomyosarcoma, small cell lung cancer and other diseases), including immunotherapy (31), overcoming drug resistance (35), trials of new combinations of antitumor drugs (43), clinical trials in young and elderly tumor patients (46, 47) and electric field treatment of tumors (40), will further advance the field of TMZ in the next 20 years.

Notably, in the current state of the COVID-19 pandemic, there is a considerable amount of literature addressing TMZ and COVID-19. According to studies, TMZ-associated immunosuppression increased mortality from severe acute respiratory syndrome coronavirus 2 (SARS-CoV-2) virus infection significantly during the COVID-19 outbreak (64–66). This study demonstrates the neurophilic potential of the SARS-CoV-2 virus and may also guide the clinical management of glioblastoma patients during the new coronary pneumonia pandemic.

The following recommendations are made based on the findings of this study: To begin, this section requires a significant amount of basic research and clinical trials in the combination of TMZ with other drugs, and although there are many challenges, they cannot be ignored. Second, researchers must identify the particular research value of antineoplastic medications (including TMZ) acting in young children and elderly cancer patients. Therefore, future drug studies targeting TMZ need to be conducted in adult cancer patients aged 18-60 years and young children and elderly cancer patients as an integral part of the research. Third, to encourage the invention of new therapies such as tumor electric field therapy to facilitate the association between new therapies and TMZ, there is a need to enhance multidisciplinary and multidisciplinary communication and integration for the treatment and diagnosis of refractory oncologic diseases.

## Conclusion

This review uses bibliometric methods to provide a systematic overview of the research process, research hotspots, and research development directions of TMZ during 1994-2021. This research will help researchers better understand the current research status and development trend of TMZ worldwide.

## Author contributions

XR and X-JF conceived and designed the article; XR, PS, KX, HL, and Z-WL collected data; XR and PS wrote the original draft; X-JF and XR edited and reviewed the paper. All authors contributed to the article and approved the submitted version.

## Funding

This study was funded by the National Natural Science Foundation of China (Grant Nos. 41806191 and 81473369) and Key R & D project of Shandong Province (Grant No. 2016CYJS08A01-1).

## Conflict of interest

The authors declare that the research was conducted in the absence of any commercial or financial relationships that could be construed as a potential conflict of interest.

## Publisher’s note

All claims expressed in this article are solely those of the authors and do not necessarily represent those of their affiliated organizations, or those of the publisher, the editors and the reviewers. Any product that may be evaluated in this article, or claim that may be made by its manufacturer, is not guaranteed or endorsed by the publisher.
